# Epigenetic Approach to PTSD: In the Aspects of Rat Models

**DOI:** 10.1055/s-0041-1736633

**Published:** 2021-11-11

**Authors:** Asli Aykac, Rasime Kalkan

**Affiliations:** 1 Department of Biophysics, Faculty of Medicine, Near East University, Nicosia, Cyprus; 2 Department of Molecular Biology and Genetics, Faculty of Arts and Sciences, Near East University, Nicosia, Cyprus; 3 Department of Medical Genetics, Faculty of Medicine, Near East University, Nicosia, Cyprus

**Keywords:** PTSD, epigenetics, methylation, rat studies

## Abstract

Posttraumatic stress disorder (PTSD) is a stress-related mental disorder and develops after exposure to life-threatening traumatic experiences. The risk factors of PTSD included genetic factors; alterations in hypothalamic–pituitary–adrenal (HPA) axis; neurotrophic, serotonergic, dopaminergic, and catecholaminergic systems; and a variety of environmental factors, such as war, accident, natural disaster, pandemic, physical, or sexual abuse, that cause stress or trauma in individuals. To be able to understand the molecular background of PTSD, rodent animal models are widely used by researchers. When looking for a solution for PTSD, it is important to consider preexisting genetic risk factors and physiological, molecular, and biochemical processes caused by trauma that may cause susceptibility to this disorder. In studies, it is reported that epigenetic mechanisms play important roles in the biological response affected by environmental factors, as well as the task of programming cell identity. In this article, we provided an overview of the role of epigenetic modifications in understanding the biology of PTSD. We also summarized the data from animal studies and their importance during the investigation of PTSD. This study shed light on the epigenetic background of stress and PTSD.

## Introduction

Posttraumatic stress disorder (PTSD), which is among the stress-related mental disorders, develops after exposure to life-threatening traumatic experiences. The main risk factor of PTSD is genetic factors which are affected by many important biological systems, such as the hypothalamic–pituitary–adrenal (HPA) axis; neurotrophic, serotonergic, dopaminergic, and catecholaminergic systems; and a variety of environmental factors, such as war, accident, natural disaster, pandemic, physical or sexual abuse, that cause stress or trauma in individual. 1 2 3

Exposure to trauma varies from person to person, depending on many interacting risk factors, including gender, genetics, and social interaction, and early life experiences. 4 5 As a result of this difference, the individual may not develop PTSD, or the symptoms seen in people who develop PTSD may spontaneously improve or, on the contrary, the individual may be at high risk of recurrence of symptoms, even though they have recovered from PTSD. 6 The fact that some individuals are still in the high-risk group for PTSD despite the decrease in symptoms emphasizes that the effects of long-term processes should be taken into account in the response to trauma. Therefore, when seeking a solution for PTSD, it is important to consider preexisting genetic risk factors and physiological, molecular, and biochemical processes caused by trauma that may cause susceptibility to this disorder. Although it is an effective method to use patients while investigating human diseases, it is not ethically appropriate to induce PTSD in healthy individuals and the occurrence of mental disorders such as PTSD in humans is not always real-time but often coincidental. 7 Since of all these cases that limit the use of human subjects, rodent animal models are generally accepted and used by research groups in PTSD studies.

## Existing Rodent Models of Inducing Symptoms Seen in PTSD

Present animal models fall into three different categories to induce symptoms seen in people with PTSD; it uses physical, social, and psychological stress factors, and these stress factors can reveal common or differentiated neuroadaptation patterns in all models. 8 In physical stress administrations, different stressors are used either alone or in combination to evaluate behavioral responses to stress. The time-dependent sensitization model which is based on sensitivity, mainly uses psychological or pharmacological stressors. The common feature of all these experiments is sensitization. A weak stimulus given after a dangerous stimulus causes an increase in sensitivity, although it is less than the intensity of the life-threatening stimulus. Under natural conditions, sensitization is a principal action to threat as it accelerates escape responses and thus may protect the subject from future danger. 9 Underwater trauma model is based on forced to swim for 1 minute, then held underwater during 30 seconds. Foot shock is a model in which the electric shock (in different intensity and duration) applied to the foot of the animal placed in the apparatus is used as a physical factor. 10 With the foot shock model, avoidance and anxiety behavior can reproduce some of the main symptoms of PTSD, including hyperarousal, aggression and flashback, and sleep disturbance. 10 11 Several studies are used auditory or visual stimuli (situational reminder [SR]) in addition to the classical fear conditioning procedure such as foot shock, using these stimuli given with shock to animals to remember fear aftershock. Unlike the classical model, in the Stress-Enhanced Fear Learning (SELF) model, animals are either not exposed to any shock or are exposed to unpredictable foot shocks many times in a single session. 12 One of the most widely used PTSD models is the single prolonged stress (SPS) model. In this model, rats are exposed to varying stress factors. In the general SPS protocol, the animal is kept in a restriction cage for 2 hours, then subjected to forced swimming and exposed to diethyl ether until unconscious. 13 The restraint/immobilization stress model, also used as part of the SPS procedure, is applied by restraining the animal using plastic retention tubes or immobilizing the four limbs and head on a board in a prone position. 14 It is also possible to benefit from the natural social behaviors of animals with social stress factors, instead of directly stressful stimulants. Since humans are known to develop PTSD in response to social experiences, such as rape and childhood abuse, it is reasonable to think that this applies to another species as well.

PTSD models which are using social stressors are often used in conjunction with other PTSD psychological models. The housing instability (HI) model is applied by placing the animal randomly in cages with different animals each time. 15 This model makes sense considering that PTSD patients are affected by HI. 16 Animals subjected to this model are generally exposed to the psychosocial stress model at first. As a psychosocial stress model, predator-based models are generally used. The predator-based model is based on the hypothesis that a synergistic effect develops due to the interaction of risk factors and PTSD. Applied at different times and it includes exposure to predator stressor, random cage change, and immobilization stress application procedures. 15 17 In predator-based models, animals are exposed to either the predator itself or a clue of the predator, such as urine, faces, or feathers. Studies have shown that rats have a strong innate fear of a predator. 18 There are many studies in the literature in which trimethylthiazolin (TMT), a synthetic compound isolated from other predator odors and urine, is used instead of living animals. 19 The Predator Scent Stress is the model in which animals are exposed to the scent of their natural predator to model how humans reproduce variations in response to trauma. For example, stress group rats are exposed to dirty contaminated cat litter for 10 minutes, while rats in the control group are exposed only to clean litter for the same period. On the last day when the behavioral parameters of the animals will be evaluated with a maze (elevated plus, T or radial) or/and open field test mechanisms, animals in all groups (both stress and control groups) are exposed to clean cat litter for 10 minutes, creating a situation that reminds trauma and reexperiencing trauma. 20

Recall of traumatic memories, which are among the symptoms of PTSD, is directly related to dysregulation and deterioration in the reconstruction of fear memory. 21 Moreover, traumatic events can also affect memory formation as they often marked strong memories. Therefore, PTSD models which using fear conditioning is focused on the processing of fear memory, behavioral responses, physiological, molecular, and neuro epigenetic processes in the basic brain regions responsible for fear learning and memory processes such as the hippocampus, amygdala, and frontal cortex. 21 22 23

After different combinations of procedures, animals display impaired habituation behaviors in their new environment. 24 It is also possible to induce PTSD-like symptoms using social isolation and maternal separation (early life stress model) such as the random cage change made in the HI model. 25 26 In the social defeat-SD model, which is used to trigger-off avoidance behavior among PTSD symptoms, subjects are exposed to a single aggressive animal. 27 28 Although both physical and social stress factors produce behavioral and neuroendocrine changes observed in PTSD, most of the relevant models do not take into account that people show different responses to trauma, some individuals are sensitive to PTSD, and some are resistant. Psychological stress, which elicits instinctive responses in animals to natural predators, more successfully reveals the difference between those susceptible to the development of PTSD and resistant ones. 7

The models that follow the provocation of fear and stress in animals are based on the elimination of the stress factor and the comparison of recovery and related biological responses with homeostasis. Animal models of PTSD can mimic human symptoms quite well, depending on the conditioning or triggering stressor factor. Unfortunately, animal models cannot explain well the differences between individuals in fear acquisition and disappearance of fear. 29 30

PTSD models that expose animals to the factors of trauma before fear conditioning may be said to have greater validity than fear conditioning alone. To be able to determine the epigenetic effect of trauma and stress subsequently, fear-learning stress and/or trauma-enhanced fear learning models has been used. These studies have been provided to define the important role of drain-derived neurotrophic factor (BDNF) on stress and fear memory. According to the results of the SEFL paradigm study, the first response is included the hippocampal BDNF/TrkB signal to fear to condition which increases exon I and IV BDNF mRNA levels, acetylation in H3 and H4 histones, and TrkB protein levels in the exon I and IV BDNF promoter regions. BDNF, which is considered to be a key regulator of the development of neurons in the central nervous system, is thought to be an important factor mediating synaptic plasticity. 31 32

There is accumulating evidence in the literature that traumatic experiences are associated with epigenetic modifications in the human genome. 33 Epigenetic modifications are associated with alteration in gene expression. Also, the epigenome is one of the susceptibility factors which contributes to PTSD. 33 34

## Epigenetic Alterations in Posttraumatic Stress Disorder

Genetic and environmental factors play a key role during the development of PTSD. Advancement of molecular and genetic diagnostic methods help identification of genetic alterations of PTSD. 34 Studies highlighted that parental exposure to posttraumatic stress can cause potential biological alterations in their offspring. 35 36 These studies show the importance of the heritability of PTSD and paved the way for genetic studies. Clinical diagnosis of PTSD is based on behavioral symptom clusters that are most directly associated with brain function, epigenetic studies of PTSD in humans have been limited to peripheral tissues such as blood, buccal tissue, and saliva. Animal models of PTSD, predominantly in rodents, have been used for the identification of the epigenetic alterations in the brain.

Epigenetic modifications are effects on gene expression status without altering nucleotide sequence. Epigenetic modifications involve DNA methylation, histone modifications, RNA modifications, and noncoding RNAs. Epigenetic alterations are influenced by both environmental and genetic factors. Epigenetic alterations are under control by several enzymes, like DNA methyltransferases, DNA demethylases, lysine methyltransferases and demethylases, and so on. The reversible nature of the epigenetic alterations is the crucial point of these modifications, and this makes them a potential target during the evaluation of disease and as a target for the treatment planning.

### Histone Modification

Histone proteins are important during the packaging of DNA and directly related to gene regulation. Histones chemically modified through the action of enzymes to regulate gene transcription. Histone acetyltransferase (HAT) is responsible for histone acetylation and acetylation of histones causes the noncondensing or loosening of DNA which allows transcription. Histone deacetylase (HDAC) is responsible to remove the acetyl group from histones and causes the coiling of DNA, creating condensed densely packed DNA which inhibits or repress the gene expression. Histone modifications are attached N-terminal tail of histones covalently. These modifications are involved acetylation, methylation, phosphorylation, ubiquitination, and sumoylation. 37 Lubin and Sweatt demonstrated that a global increase in histone H3 acetylation and phosphorylation was related to recalling of fear memory in the CA1-4 region of the hippocampus 38 and Maddox and Schafe showed recalling fear memory promoted increased level acetylation of histone H3 in the lateral amygdala. 39 In a study using a rodent model of PTSD, it was shown that histone hyperacetylation was triggered in LA after ingestion of fear memory inhibitors. 40

Studies demonstrated strong interaction between fear and histone acetylation. 41 42 Webb et al demonstrated the interaction between fear memory and H3K4me3 and 5-hydroxymethylcytosine (5-hmc) in the hippocampal CA1 region and anterior cingulate cortex (ACC). 42 Therefore, these studies show us the interaction between epigenetic alterations and their effects on different brain region during fear memory formation. Takei et al used an SEFL paradigm with an SPS in rats and observed an increased level of acetylation of histone H3 and H4 at the exon I and IV BDNF promoter regions in SPS rats in their hippocampus. 43 Takei et al demonstrated enhanced hippocampal BDNF/TrkB signaling in response to fear conditioning in this animal model of PTSD. 43 The reversible nature of epigenetic modifications makes them a therapeutic target. HDAC, DNA Methyltransferases (DNMT), and Histone methyltransferases (HMT) inhibitors are widely used inhibitors in clinical practice and researches which changes the effects of modifications especially in cancer. Studies emphasized that drugs targeting HDACs and DNA methyltransferase inhibitors as an emerging anticancer strategy and The Food and Drug Administration (FDA) has approved these agents as a treatment target. Lee et al demonstrated that knockdown of HDAC6 prevents the enhancement of glutamatergic signaling by acute stress in rats. 44 Studies demonstrated that HDAC inhibitors sodium butyrate (NaB), trichostatin A, and valproic acid which elevate histone acetylation levels, has therapeutic effects in rodent stress paradigms, improving cognition, and reducing anxiety behaviors. 45

### DNA Methylation

DNA methylation is one of the most studied epigenetic alterations. Methyl groups bind covalently fifth carbon of cytosine nucleotides on DNA. DNA methylation is a reversible process and DNA methyltransferases are responsible for adding methyl groups and DNA demethylases are responsible for removing those methyl groups. Methylation of cytosine nucleotide is directly related to gene expression. DNA methylation is important during tissue differentiation, tissue-specific gene expression, and X inactivation. 46 Advancement of diagnostic tools will help identification of different DNA modifications, like 5-methylcytosine (5-mc) and 5-hmc. 37 47 These modifications are important in PTSD and learning.

Methylation studies showed that FK506 binding protein 51 gene, *BRSK1* , *LCN8* , *NFG* , *DOCK2* , *ZFP57* , *RNF39* , 46 47 48 *BDNF* , *NR3C1* , *MAN2C1* , *TLR8* , *SLC6A4* , *IL-18* , *SKA2* , 3 *LINC01090, BC036345, ZNRD1-ASI* , and *RORA* , 49 50 51 52 53 54 55 *TPR* , *ANXA2* , *CLEC9A* , *ACP5* , *TLR8* , *LRRC3B* , *BRSK1* , *LCN8* , *NGF* , *DOCK2* , 48 *DUSP22* , *HIST1H2APS2* , *HOOK2* , *NINJ2* , *PAX8* , *RNF39* , *ZFP57* , 56 *NRG1* , *HGS* genes are related with PTSD and severity of symptoms 48 56 57 ( Table 1 ). The FK506-binding protein 51 ( *FKBP5* ) gene is one of the genes on HPA axis and an important regulator of stress response through altering glucocorticoid receptor sensitivity. 58 Klengel et al identified an interaction between demethylation of FKBP5 and early trauma exposure. 55 Rodent studies demonstrated that parental care influences revealed that differences in maternal phenotypes effects their pup's development of behavioral and HPA responses to stress as adults. 59 Elevated level DNA methylation was identified in the offspring after low maternal care and lower methylation was identified in high maternal care. 59 60 Li et al demonstrated the interaction between fear extinction and Tet3-mediated global 5-hmc in the infralimbic region of the ventromedial prefrontal cortex. 61

**Table 1:** Differentially methylated human genes in PTSD

Gene	Tissue type	Species	Methylation status	Reference
*BRSK1*	Blood	Human	Hyper	46–48
*LCN8*	Blood	Human	Hyper	46–48
*NFG*	Blood	Human	Hyper	46–48
*DOCK2*	Blood	Human	Hyper	46–48
*ZFP57*	Blood	Human	Hyper	46–48
*RNF39*	Blood	Human	Hyper	46–48
*NR3C1*	Whole blood	Human	Hypo	3
*MAN2C1*	Whole blood	Human	Hypo	3
*TLR8*	Whole blood	Human	Hypo	3
*SLC6A4*	Whole blood	Human	Hypo	3
*IL-18*	Whole blood	Human	Hypo	3
*SKA2*	Whole blood	Human	Hypo	3
*DUSP22*	Blood	Human	NS	49
*HIST1H2APS2*	Blood	Human	Hypo	49
*HOOK2*	Blood	Human	NS	49
*NINJ2*	Blood	Human	NS	49
*PAX8*	Blood	Human	NS	49
*RNF39*	Blood	Human	Hypo	49
*ZFP57*	Blood	Human	Hypo	49

Abbreviations: Hyper, hypermethylation in case relative to control; Hypo, hypomethylation in case relative to control; NS, nonspecified; PTSD, posttraumatic stress disorder.

Studies highlighted that different brain regions, like dentate gyrus (DG), dorsal DG, dorsal CA1, ventral CA3, basolateral amygdala, and medial prefrontal cortex, show different level of gene expression during the stress induction. 62 Roth et al reported that in PTSD, BDNF exon IV was hypomethylated in the ventral CA3 and no methylation changes observed in other hippocampal subregions in rats. 63 It is also reported in the literature that there is a relationship between the epigenetic status of catechol-O-methyltransferase and PTSD. 64

*NR3C1* is another most studied gene which encodes the GR. *NR3C1* plays a key role in the understanding of the epigenetic regulation of PTSD and fear extinction processes. 65 As a result of the study examining the relationship between the methylation level of the *NR3C1* promoter and PTSD, it is emphasized that the higher level of DNA methylation in the NR3C1 promoter region could be a protective effect against vulnerability to PTSD. 66

*HDAC4* gene was related to learning and memory-related processes and studies demonstrated that higher level methylation was observed in PTSD cases. 63 In the study where *BDNF* , *NR3C1* , *MAN2C1* , *TLR8* , *SLC6A4* , *IL-18* , and *SKA2* gene methylation analyses were performed in patients suffering from PTSD, it was concluded that BDNF, NR3C1 and MAN2C1 methylations are associated with PTSD diagnosis. 3 This study revealed the importance the regulation of genes involved in synaptic plasticity and the HPA axis and their association with PTSD.

### Noncoding RNA

Noncoding RNAs (ncRNAs) are not translated into a polypeptide. They play a key role during the processing and regulation of other RNAs such as messenger RNA (mRNA), transfer RNA (tRNA), and ribosomal RNA (rRNA). 67 68 Recent studies showed the importance of ncRNAs as a biomarker for trauma-related brain disorders. 69 Ambeskovic et al demonstrated how transgenerational and multigenerational stress factors regulate cortical miR-221 and miR-26 expression and their target genes, ntrk2, and map1a, and crh in the rat. They concluded that early life experiences and prenatal stress are crucial factors for brain development and mental health. 70

Maurel et al studied miR-15a-5p, miR-497a-5p, miR-511–5p, and let-7d-5p in brain areas of mouse model of PTSD. 71 They identified lower transcript levels of miR-15a-5p, miR-497a-5p, and miR-511a-5p in the hippocampus and hypothalamus and in the medial prefrontal cortex, downregulation of miR-15a-5p, miR-511–5p, and let-7d-5p. They concluded that miRNA expression in the different brain areas correlated to miRNA-based epigenetic modulation in stress-induced phenotypes. 71

Li et al demonstrated that exposure of acute traumatic stress in early adolescent caused permanent changes in neural network and altered expression of CRFR1 and CRFR1 mRNA and miR-34c expression in Wistar rats. 72 These studied showed the importance of identification novel non coding RNAs as a candidate for biomarkers of PTSD.

## Conclusion

PTSD characterized by systemic dysregulation in anatomical sites outside of the brain, maladaptive alterations of the HPA axis, and sympathetic nervous system sensitivity/responsivity, and alterations in neuroimmune dynamics. 59 Human-based epigenetic studies of PTSD demonstrated alterations in age-related CpGs, DNA methylation, alteration in estrogen-responsive genes, alterations in the dopaminergic system, and alterations in the HPA axis. Environmental factors may have long-term consequences on brain behavior as a result of interaction with the genome by regulating epigenetic mechanisms. The susceptibility of genes due to stress and/or trauma, the effect of epigenetic changes on the development of PTSD has recently become more remarkable. In the literature, methylation analyses were performed using genes encoding proteins, such as BDNF, FKBP5, IL-18, MAN2C1, NR3C1, SKA2, SLC6A4 and TLR8, which play a role in the synaptic plasticity, immune system, serotonin modulation, neurogenesis, and inflammation response in PTSD. 3 73 While clinical researches are vital in the administration of new therapeutic agents to people with PTSD, animal models are indispensable for comparing the behavioral and physiological effects caused by trauma, as well as molecular and biochemical abnormalities and the state of homeostasis.

Epigenetics studies provide a crucial role in understanding the interaction of epigenetics with environmental exposure to trauma in PTSD. Further studies will shed light on the interaction between stress-induced epigenetic regulations of neuronal function. The reversible nature of epigenetic modifications promises for determination of the epigenetic architecture of PTSD and may play a crucial role during the discovery of novel targeted therapeutic approaches to develop treatment strategies and prevention of PTSD. These studies will shed light on resolving several issues, like epigenetic treatment, trauma, or stress memory. Studies in the field of epigenetics will help to discover a marker for susceptibility to PTSD.

## References

[1] Gradus J L Qin P Lincoln A K Posttraumatic stress disorder and completed suicide Am J Epidemiol 2010 171 06 721 727 20160171 10.1093/aje/kwp456

[2] Forte G Favieri F Tambelli R Casagrande M COVID-19 pandemic in the Italian population: validation of a post-traumatic stress disorder questionnaire and prevalence of PTSD symptomatology Int J Environ Res Public Health 2020 17 11 4151 4164 10.3390/ijerph17114151 PMC7312976 32532077

[3] Hossack M R Reid M W Aden J K Gibbons T Noe J C Willis A M Adverse childhood experience, genes, and PTSD risk in soldiers: a methylation study Mil Med 2020 185(3-4):377 384 32976583 10.1093/milmed/usz292

[4] Zoladz P R Diamond D M Predator-based psychosocial stress animal model of PTSD: Preclinical assessment of traumatic stress at cognitive, hormonal, pharmacological, cardiovascular and epigenetic levels of analysis Exp Neurol 2016 284(pt. B):211 219 27283115 10.1016/j.expneurol.2016.06.003

[5] Voisey J Young R M Lawford B R Morris C P Progress towards understanding the genetics of posttraumatic stress disorder J Anxiety Disord 2014 28 08 873 883 25445077 10.1016/j.janxdis.2014.09.014

[6] Solomon Z Mikulincer M Trajectories of PTSD: a 20-year longitudinal study Am J Psychiatry 2006 163 04 659 666 16585441 10.1176/ajp.2006.163.4.659

[7] Borghans B Homberg J R Animal models for posttraumatic stress disorder: An overview of what is used in research World J Psychiatry 2015 5 04 387 396 26740930 10.5498/wjp.v5.i4.387 PMC4694552

[8] Warren B L Vialou V F Iñiguez S D Neurobiological sequelae of witnessing stressful events in adult mice Biol Psychiatry 2013 73 01 7 14 22795644 10.1016/j.biopsych.2012.06.006 PMC3498570

[9] Antelman S M Time-dependent sensitization as the cornerstone for a new approach to pharmacotherapy - drugs as foreign stressful stimuli Drug Dev Res 1988 14 1 30

[10] Louvart H Maccari S Ducrocq F Thomas P Darnaudéry M Long-term behavioural alterations in female rats after a single intense footshock followed by situational reminders Psychoneuroendocrinology 2005 30 04 316 324 15694111 10.1016/j.psyneuen.2004.09.003

[11] Pawlyk A C Jha S K Brennan F X Morrison A R Ross R J A rodent model of sleep disturbances in posttraumatic stress disorder: the role of context after fear conditioning Biol Psychiatry 2005 57 03 268 277 15691528 10.1016/j.biopsych.2004.11.008

[12] Rau V Fanselow M S Exposure to a stressor produces a long lasting enhancement of fear learning in rats Stress 2009 12 02 125 133 18609302 10.1080/10253890802137320

[13] Liberzon I Krstov M Young E A Stress-restress: effects on ACTH and fast feedback Psychoneuroendocrinology 1997 22 06 443 453 9364622 10.1016/s0306-4530(97)00044-9

[14] Balkaya M Prinz V Custodis F Stress worsens endothelial function and ischemic stroke via glucocorticoids Stroke 2011 42 11 3258 3264 21921276 10.1161/STROKEAHA.110.607705

[15] Zoladz P R Conrad C D Fleshner M Diamond D M Acute episodes of predator exposure in conjunction with chronic social instability as an animal model of post-traumatic stress disorder Stress 2008 11 04 259 281 18574787 10.1080/10253890701768613 PMC2535807

[16] Kim H G Harrison P A Godecker A L Muzyka C N Posttraumatic stress disorder among women receiving prenatal care at three federally qualified health care centers Matern Child Health J 2014 18 05 1056 1065 23912314 10.1007/s10995-013-1333-7

[17] Zoladz P R Fleshner M Diamond D M Psychosocial animal model of PTSD produces a long-lasting traumatic memory, an increase in general anxiety and PTSD-like glucocorticoid abnormalities Psychoneuroendocrinology 2012 37 09 1531 1545 22421563 10.1016/j.psyneuen.2012.02.007

[18] Blanchard R J Blanchard D C Rodgers J Weiss S M The characterization and modelling of antipredator defensive behavior Neurosci Biobehav Rev 1990 14 04 463 472 2287483 10.1016/s0149-7634(05)80069-7

[19] Apfelbach R Blanchard C D Blanchard R J Hayes R A McGregor I S The effects of predator odors in mammalian prey species: a review of field and laboratory studies Neurosci Biobehav Rev 2005 29 08 1123 1144 16085312 10.1016/j.neubiorev.2005.05.005

[20] Cohen H Geva A B Matar M A Zohar J Kaplan Z Post-traumatic stress behavioural responses in inbred mouse strains: can genetic predisposition explain phenotypic vulnerability? Int J Neuropsychopharmacol 2008 11 03 331 349 17655807 10.1017/S1461145707007912

[21] Careaga M BL Girardi C EN Suchecki D Understanding posttraumatic stress disorder through fear conditioning, extinction and reconsolidation Neurosci Biobehav Rev 2016 71 48 57 27590828 10.1016/j.neubiorev.2016.08.023

[22] Zovkic I B Sweatt J D Epigenetic mechanisms in learned fear: implications for PTSD Neuropsychopharmacology 2013 38 01 77 93 22692566 10.1038/npp.2012.79 PMC3521992

[23] Kwapis J L Wood M A Epigenetic mechanisms in fear conditioning: implications for treating post-traumatic stress disorder Trends Neurosci 2014 37 12 706 720 25220045 10.1016/j.tins.2014.08.005 PMC4258434

[24] Saavedra-Rodríguez L Feig L A Chronic social instability induces anxiety and defective social interactions across generations Biol Psychiatry 2013 73 01 44 53 22906514 10.1016/j.biopsych.2012.06.035 PMC3826464

[25] Imanaka A Morinobu S Toki S Yamawaki S Importance of early environment in the development of post-traumatic stress disorder-like behaviors Behav Brain Res 2006 173 01 129 137 16860405 10.1016/j.bbr.2006.06.012

[26] Cloitre M Stolbach B C Herman J L A developmental approach to complex PTSD: childhood and adult cumulative trauma as predictors of symptom complexity J Trauma Stress 2009 22 05 399 408 19795402 10.1002/jts.20444

[27] Pulliam J V Dawaghreh A M Alema-Mensah E Plotsky P M Social defeat stress produces prolonged alterations in acoustic startle and body weight gain in male Long Evans rats J Psychiatr Res 2010 44 02 106 111 19573876 10.1016/j.jpsychires.2009.05.005 PMC2813344

[28] Yang R Daigle B J Jr. Muhie S Y Core modular blood and brain biomarkers in social defeat mouse model for post traumatic stress disorder BMC Syst Biol 2013 7 80 23962043 10.1186/1752-0509-7-80 PMC3751782

[29] Ursano R J Li H Zhang L Models of PTSD and traumatic stress: the importance of research “from bedside to bench to bedside” Prog Brain Res 2008 167 203 215 18037016 10.1016/S0079-6123(07)67014-9

[30] Yehuda R Hoge C W McFarlane A C Post-traumatic stress disorder Nat Rev Dis Primers 2015 1 15057 27189040 10.1038/nrdp.2015.57

[31] Frielingsdorf H Bath K G Soliman F Difede J Casey B J Lee F S Variant brain-derived neurotrophic factor Val66Met endophenotypes: implications for posttraumatic stress disorder Ann N Y Acad Sci 2010 1208 150 157 20955337 10.1111/j.1749-6632.2010.05722.x PMC3032081

[32] Matsuoka Y Nishi D Noguchi H Kim Y Hashimoto K Longitudinal changes in serum brain-derived neurotrophic factor in accident survivors with posttraumatic stress disorder Neuropsychobiology 2013 68 01 44 50 23774996 10.1159/000350950

[33] Maddox S A Watts C S Doyère V Schafe G E A naturally-occurring histone acetyltransferase inhibitor derived from Garcinia indica impairs newly acquired and reactivated fear memories PLoS One 2013 8 01 e54463 23349897 10.1371/journal.pone.0054463 PMC3549978

[34] Sartor C E McCutcheon V V Pommer N E Common genetic and environmental contributions to post-traumatic stress disorder and alcohol dependence in young women Psychol Med 2012 41 07 1497 1505 10.1017/S0033291710002072 PMC3377473 21054919

[35] Leen-Feldner E W Feldner M T Knapp A Bunaciu L Blumenthal H Amstadter A B Offspring psychological and biological correlates of parental posttraumatic stress: review of the literature and research agenda Clin Psychol Rev 2013 33 08 1106 1133 24100080 10.1016/j.cpr.2013.09.001

[36] Lambert J E Holzer J Hasbun A Association between parents' PTSD severity and children's psychological distress: a meta-analysis J Trauma Stress 2014 27 01 9 17 24464491 10.1002/jts.21891

[37] Basavarajappa B S Subbanna S Histone methylation regulation in neurodegenerative disorders Int J Mol Sci 2021 22 09 4654-4681. 33925016 10.3390/ijms22094654 PMC8125694

[38] Lubin F D Sweatt J D The IkappaB kinase regulates chromatin structure during reconsolidation of conditioned fear memories Neuron 2007 55 06 942 957 17880897 10.1016/j.neuron.2007.07.039 PMC2587178

[39] Maddox S A Schafe G E Epigenetic alterations in the lateral amygdala are required for reconsolidation of a Pavlovian fear memory Learn Mem 2011 18 09 579 593 21868438 10.1101/lm.2243411 PMC3166787

[40] Maddox S A Watts C S Schafe G E p300/CBP histone acetyltransferase activity is required for newly acquired and reactivated fear memories in the lateral amygdala Learn Mem 2013 20 02 109 119 23328899 10.1101/lm.029157.112 PMC3549061

[41] Stafford J M Raybuck J D Ryabinin A E Lattal K M Increasing histone acetylation in the hippocampus-infralimbic network enhances fear extinction Biol Psychiatry 2012 72 01 25 33 22290116 10.1016/j.biopsych.2011.12.012 PMC3352991

[42] Webb W M Sanchez R G Perez G Dynamic association of epigenetic H3K4me3 and DNA 5hmC marks in the dorsal hippocampus and anterior cingulate cortex following reactivation of a fear memory Neurobiol Learn Mem 2017 142(pt. A):66 78 28232238 10.1016/j.nlm.2017.02.010

[43] Takei S Morinobu S Yamamoto S Fuchikami M Matsumoto T Yamawaki S Enhanced hippocampal BDNF/TrkB signaling in response to fear conditioning in an animal model of posttraumatic stress disorder J Psychiatr Res 2011 45 04 460 468 20863519 10.1016/j.jpsychires.2010.08.009

[44] Lee J B Wei J Liu W Cheng J Feng J Yan Z Histone deacetylase 6 gates the synaptic action of acute stress in prefrontal cortex J Physiol 2012 590 07 1535 1546 22331421 10.1113/jphysiol.2011.224907 PMC3413488

[45] Blouin A M Sillivan S E Joseph N F Miller C A The potential of epigenetics in stress-enhanced fear learning models of PTSD Learn Mem 2016 23 10 576 586 27634148 10.1101/lm.040485.115 PMC5026205

[46] Jin B Li Y Robertson K D DNA methylation: superior or subordinate in the epigenetic hierarchy? Genes Cancer 2011 2 06 607 617 21941617 10.1177/1947601910393957 PMC3174260

[47] Khare T Pai S Koncevicius K 5-hmC in the brain is abundant in synaptic genes and shows differences at the exon-intron boundary Nat Struct Mol Biol 2012 19 10 1037 1043 22961382 10.1038/nsmb.2372 PMC3465469

[48] Mehta D Bruenig D Carrillo-Roa T Genomewide DNA methylation analysis in combat veterans reveals a novel locus for PTSD Acta Psychiatr Scand 2017 136 05 493 505 28795405 10.1111/acps.12778

[49] Logue M W Amstadter A B Baker D G The Psychiatric Genomics Consortium Posttraumatic Stress Disorder Workgroup: posttraumatic stress disorder enters the age of large-scale genomic collaboration Neuropsychopharmacology 2015 40 10 2287 2297 25904361 10.1038/npp.2015.118 PMC4538342

[50] Almli L M Stevens J S Smith A K A genome-wide identified risk variant for PTSD is a methylation quantitative trait locus and confers decreased cortical activation to fearful faces Am J Med Genet B Neuropsychiatr Genet 2015 8 327 336 10.1002/ajmg.b.32315 PMC4844461 25988933

[51] Xie P Kranzler H R Yang C Zhao H Farrer L A Gelernter J Genome-wide association study identifies new susceptibility loci for posttraumatic stress disorder Biol Psychiatry 2013 74 09 656 663 23726511 10.1016/j.biopsych.2013.04.013 PMC3810148

[52] Duncan L E Ratanatharathorn A Aiello A E Largest GWAS of PTSD (N=20 070) yields genetic overlap with schizophrenia and sex differences in heritability Mol Psychiatry 2018 23 03 666 673 28439101 10.1038/mp.2017.77 PMC5696105

[53] Veterans Affairs Mid-Atlantic Mental Illness Research, Education, and Clinical Center Workgroup Ashley-Koch A E Garrett M E Gibson J Genome-wide association study of posttraumatic stress disorder in a cohort of Iraq-Afghanistan era veterans J Affect Disord 2015 184 184 225 234 26114229 10.1016/j.jad.2015.03.049 PMC4697755

[54] Kilaru V Iyer S V Almli L M Genome-wide gene-based analysis suggests an association between neuroligin 1 (NLGN1) and post-traumatic stress disorder Transl Psychiatry 2016 6 05 e820 27219346 10.1038/tp.2016.69 PMC5070067

[55] Department of Veterans Affairs Cooperative Studies Program (#575B) and Million Veteran Program Gelernter J Sun N Polimanti R Genome-wide association study of post-traumatic stress disorder reexperiencing symptoms in >165,000 US veterans Nat Neurosci 2019 22 09 1394 1401 31358989 10.1038/s41593-019-0447-7 PMC6953633

[56] Rutten B PF Vermetten E Vinkers C H Longitudinal analyses of the DNA methylome in deployed military servicemen identify susceptibility loci for post-traumatic stress disorder Mol Psychiatry 2018 23 05 1145 1156 28630453 10.1038/mp.2017.120 PMC5984086

[57] Hammamieh R Chakraborty N Gautam A Whole-genome DNA methylation status associated with clinical PTSD measures of OIF/OEF veterans Transl Psychiatry 2017 7 07 e1169 28696412 10.1038/tp.2017.129 PMC5538114

[58] van Dongen J Slagboom P E Draisma H HM Martin N G Boomsma D I The continuing value of twin studies in the omics era Nat Rev Genet 2012 13 09 640 653 22847273 10.1038/nrg3243

[59] Zhang T Y Labonté B Wen X L Turecki G Meaney M J Epigenetic mechanisms for the early environmental regulation of hippocampal glucocorticoid receptor gene expression in rodents and humans Neuropsychopharmacology 2013 38 01 111 123 22968814 10.1038/npp.2012.149 PMC3521971

[60] Klengel T Mehta D Anacker C Allele-specific FKBP5 DNA demethylation mediates gene-childhood trauma interactions Nat Neurosci 2013 16 01 33 41 23201972 10.1038/nn.3275 PMC4136922

[61] Li X Wei W Zhao Q Y Neocortical Tet3-mediated accumulation of 5-hydroxymethylcytosine promotes rapid behavioral adaptation Proc Natl Acad Sci U S A 2014 111 19 7120 7125 24757058 10.1073/pnas.1318906111 PMC4024925

[62] Kim G S Smith A K Nievergelt C M Uddin M Neuroepigenetics of Post-Traumatic Stress Disorder Prog Mol Biol Transl Sci 2018 158 227 253 30072055 10.1016/bs.pmbts.2018.04.001 PMC6474244

[63] Roth T L Zoladz P R Sweatt J D Diamond D M Epigenetic modification of hippocampal Bdnf DNA in adult rats in an animal model of post-traumatic stress disorder J Psychiatr Res 2011 45 07 919 926 21306736 10.1016/j.jpsychires.2011.01.013 PMC3335738

[64] Gillespie C F Bradley B Mercer K Trauma exposure and stress-related disorders in inner city primary care patients Gen Hosp Psychiatry 2009 31 06 505 514 19892208 10.1016/j.genhosppsych.2009.05.003 PMC2785858

[65] de Quervain D J Aerni A Schelling G Roozendaal B Glucocorticoids and the regulation of memory in health and disease Front Neuroendocrinol 2009 30 03 358 370 19341764 10.1016/j.yfrne.2009.03.002

[66] Yehuda R Daskalakis N P Desarnaud F Epigenetic biomarkers as predictors and correlates of symptom improvement following psychotherapy in combat veterans with PTSD Front Psychiatry 2013 4 118 24098286 10.3389/fpsyt.2013.00118 PMC3784793

[67] Collins L J Schönfeld B Chen X S The epigenetics of non-coding RNA San Diego, CA Academic Press 2011 49 61

[68] Qureshi I A Mehler M F Emerging roles of non-coding RNAs in brain evolution, development, plasticity and disease Nat Rev Neurosci 2012 13 08 528 541 22814587 10.1038/nrn3234 PMC3478095

[69] Daskalakis N P Rijal C M King C Huckins L M Ressler K J Recent genetics and epigenetics approaches to PTSD Curr Psychiatry Rep 2018 20 05 30 29623448 10.1007/s11920-018-0898-7 PMC6486832

[70] Ambeskovic M Babenko O Ilnytskyy Y Kovalchuk I Kolb B Metz G AS Ancestral stress alters lifetime mental health trajectories and cortical neuromorphology via epigenetic regulation Sci Rep 2019 9 01 6389 31011159 10.1038/s41598-019-42691-z PMC6476877

[71] Maurel O M Torrisi S A Barbagallo C Dysregulation of miR-15a-5p, miR-497a-5p and miR-511-5p is associated with modulation of BDNF and FKBP5 in brain areas of PTSD-related susceptible and resilient mice Int J Mol Sci 2021 22 10 5157 5177 34068160 10.3390/ijms22105157 PMC8153003

[72] Li C Liu Y Liu D Jiang H Pan F Dynamic alterations of miR-34c expression in the hypothalamus of male rats after early adolescent traumatic stress Neural Plast 2016 2016 5.249893E6 10.1155/2016/5249893 PMC4746392 26925271

[73] Kang J I Kim T Y Choi J H So H S Kim S J Allele-specific DNA methylation level of FKBP5 is associated with post-traumatic stress disorder Psychoneuroendocrinology 2019 103 1 7 30605803 10.1016/j.psyneuen.2018.12.226

